# Molecular Typing of Chikungunya Virus at a Regional Advanced Healthcare Facility in Central India: A Three-Year Prospective Study

**DOI:** 10.7759/cureus.80185

**Published:** 2025-03-06

**Authors:** Sumit K Rawat, Ram Kumar Nema, Shashwati Nema, Sudheer Gupta, Debasis Biswas

**Affiliations:** 1 Microbiology, Bundelkhand Medical College, Sagar, IND; 2 Microbiology, All India Institute of Medical Sciences, Bhopal, IND; 3 Environmental Biotechnology, Genetics and Molecular Biology, Indian Council of Medical Research (ICMR)National Institute for Research in Environmental Health Bhopal, Bhopal, IND; 4 Next Generation Sequencing (NGS) and Bioinformatics Division, 3B BlackBio Biotech India Ltd., Bhopal, IND

**Keywords:** chikungunya virus (chikv), disease outbreak, spread of virus, vector-borne diseases, whole-genome sequencing

## Abstract

Chikungunya virus (CHIKV) has emerged as a significant public health concern due to its tendency to re-emerge, causing massive outbreaks in India and globally. Recent outbreaks demonstrate the virus's ability to spread rapidly, evade the host’s immune responses, and lead to debilitating illnesses. Despite advances in public health surveillance and vector control, the cyclical, unpredictable resurgence of CHIKV underscores gaps in our understanding of its molecular dynamics and epidemiological patterns that vary by region. This study investigates the molecular and phylogenetic characteristics of CHIKV infections from 2020 to 2023 at an advanced regional tertiary care facility in Central India.

A total of 1,021 serum samples were collected from patients presenting exhibiting symptoms consistent with chikungunya infection. Of these, 178 tested positive for CHIKV IgM, and 16 were confirmed positive for CHIKV by reverse transcription-polymerase chain reaction (RT-PCR). The PCR-positive samples were then sequenced to analyze the entire viral genome. Genome annotation was performed using the Bacterial and Viral Bioinformatics Resource Center (BVBRC) database, and multiple sequence alignment (MSA) was performed using Molecular Evolutionary Genetics Analysis (MEGA) Version 11.0 (Pennsylvania State University, University Park, PA, United States).

Phylogenetic analysis revealed that the circulating strains belonged to a single clade within the East-Central-South-African (ECSA) genotype. By comparing these strains with previously reported sequences from India, we identified notable mutations in the E1 region, such as S72N, K211E, M269V, D284E, A315V, and I317V, previously found strains from Central India and New Delhi. Mutations such as M31I, I54V, and S105T, as well as the A226V mutation previously reported in India, were absent, suggesting that the currently circulating CHIKV strains in our region are primarily transmitted through *Aedes aegypti.* In contrast, mutations previously observed in the nonstructural region before 2014, such as nsP2-E145D and nsP3-V376T, re-emerged in our isolates. These findings enhance our understanding of CHIKV's genetic diversity, delineating the evolution of local CHIKV clades and their implications for regional epidemiology and public health in Central India.

## Introduction

Chikungunya fever is an acute viral illness characterized by the sudden onset of fever, chills, joint pain, and rash. The chikungunya virus (CHIKV), the causative agent, is primarily transmitted by *Aedes aegypti *and *Aedes albopictus.* It has become a major public health concern due to its tendency to re-emerge [1,2]. This recurrent issue is largely driven by the virus's ability to undergo genetic mutations, a common characteristic of RNA viruses.

Since its re-emergence in India in 2006, CHIKV has caused numerous outbreaks, leading to widespread morbidity [3-5]. Madhya Pradesh, a state in Central India, has also experienced a notable number of chikungunya cases in recent years, underscoring the need to study the virus's local genetic evolution and its potential impact on transmission dynamics and disease severity [6,7]. Understanding CHIKV's genetic diversity in this region can inform epidemiological surveillance, transmission patterns, prevention and control strategies, and vaccine development [8]. This study aims to perform whole-genome sequencing (WGS) of CHIKV strains responsible for infections in Madhya Pradesh, Central India, and compare them with previously documented sequences from other regions of India and abroad.

## Materials and methods

Sample collection

Between November 2020 and December 2023, 1,021 serum samples were collected from patients presenting with clinical symptoms suggestive of chikungunya infection at hospitals affiliated with the All India Institute of Medical Sciences (AIIMS) Bhopal. Inclusion criteria included acute onset of fever, arthralgia, and the absence of alternative diagnoses [9]. All patients provided informed consent, and the study was approved by the Institutional Review Board and the Human Ethics Committee of the institution (ref. no. 2019/PhD/Jan/19/11).

Laboratory testing

Serum samples were initially screened for anti-CHIKV IgM antibodies using the IgM antibody capture enzyme-linked immunosorbent assay (MAC ELISA) kits [10]. Samples that tested positive for IgM antibodies were then subjected to a real-time reverse transcription-polymerase chain reaction (RT-PCR), targeting the E1 gene of CHIKV to confirm active infection.

RNA extraction and RT-PCR

All the CHIKV seropositive samples stored at -80˚C were processed for RNA extraction using the QIAamp Viral RNA Mini Kit (Qiagen, Hilden, Germany) [11]. After extraction, the positive samples were tested with qRT-PCR using the TRUPCR® chikungunya RT-PCR kit (3B BlackBio Biotech India Ltd., Bhopal, India), which targets a highly conserved segment of the E1 region in the CHIKV genome [12]. The reaction mix was prepared initially with a volume of 15 µL. The PCR was performed on a Bio-Rad CFX 96 instrument for 45 cycles, with the cutoff set to 40 for detecting positive samples, according to the manufacturer's instructions. Positive RT-PCR samples with cycle threshold (Ct) values of 22 or lower were selected for sequencing.

Sequencing and phylogenetic analysis

PCR products were purified using the PCR Purification Kit (QIAquick, Qiagen, Hilden, Germany) and were quantified using Qubit (Invitrogen by Thermo Fisher Scientific, Waltham, MA, United States). Library preparation began with complementary DNA (cDNA) synthesis using the sequence-independent single-primer amplification (SISPA) technique [13]. This method allows for the amplification of cDNA without requiring a specific primer for each target sequence, making it versatile for various applications. Reverse transcription was first performed to synthesize the first strand of cDNA, followed by second strand synthesis. 

Sequencing was performed using a paired-end 2 x 150 bp run on the Illumina NovaSeq 6000 platform, generating high-quality, detailed sequence data. FastQC (V 0.12.1) software (Babraham Bioinformatics, Babraham, England) was used to assess the quality of the FASTQ sequences [14]. The generated sequence was initially analyzed using the Basic Local Alignment Search Tool (BLAST) tool (National Center for Biotechnology Information (NCBI), Bethesda, MD, United States) [15]. Editing and alignment were performed using BioEdit 7.7.1 software [16]. Genome annotation was done using the BVBRC database, and multiple sequence alignment (MSA) was conducted with Molecular Evolutionary Genetics Analysis (MEGA) version 11.0 (Pennsylvania State University, University Park, PA, United States) (using ClustalW within MEGA software) on the sequences obtained [17]. Representative sequences from major outbreaks in India, along with sequences from other countries and one Alphavirus isolate (O'nyong-nyong virus (ONNV)), were included as the out-group (see Appendix 1). Maximum likelihood phylogenetic trees were constructed using nucleotide sequences, and the reliability of the trees was tested with the bootstrap method (1000 bootstrap replications) in MEGA.

A key objective of this study was to identify mutations responsible for CHIKV's re-emergence. To achieve this, we analyzed the pattern of genetic changes at each locus to pinpoint novel mutations. We focused on two primary types of loci: unstable mutations, which tend to revert to their original form [18], and stable mutations, which continue to confer evolutionary advantages by enhancing viral fitness and persistence [19].

Statistical analysis

Data analysis was performed using the open version of GraphPad QuickCalcs (Dotmatics, Boston, MA, United States) [20]. Qualitative variables were expressed as proportions, and the chi-square test was used to assess the statistical significance of differences between two proportions. Quantitative variables, being continuous and normally distributed, were expressed as the mean and standard deviation for a direct comparison across subgroups (e.g., age groups, seasons, or years) to identify patterns in CHIKV epidemiology. Unpaired t-tests were used to evaluate the significance of differences between subgroups, with p < 0.05 considered statistically significant.

## Results

Demographic, serology, and RT-PCR findings

Among the 1,021 participants suspected of having chikungunya, 586 were male (57.4%) and 435 were female (42.6%) patients. The mean age of the patients was 29 ± 13 years (Table 1).** **

**Table 1:** Age distribution of chikungunya suspects and those who tested positive for CHIKV IgM CHIKV: chikungunya virus.

S. no.	Age group (years)	Chikungunya suspects	Chikungunya IgM positive	Positivity (%)
1	1-10	61	4	6.6
2	11-20	105	11	10.5
3	21-30	202	36	17.8
4	31-40	222	39	17.6
5	41-50	158	28	17.7
6	51-60	105	25	23.8
7	61-70	91	18	19.8
8	71-80	53	12	22.2
9	81-90	24	5	20.8
Total	-	1021	178	100

Of the 1,021 serum samples tested, 178 (17.4%) patients were IgM-positive for CHIKV, indicating a recent infection. A chi-square test was performed to assess whether IgM positivity rates varied significantly across age groups. The analysis revealed no statistically significant difference (p = 0.109).

**Figure 1:**
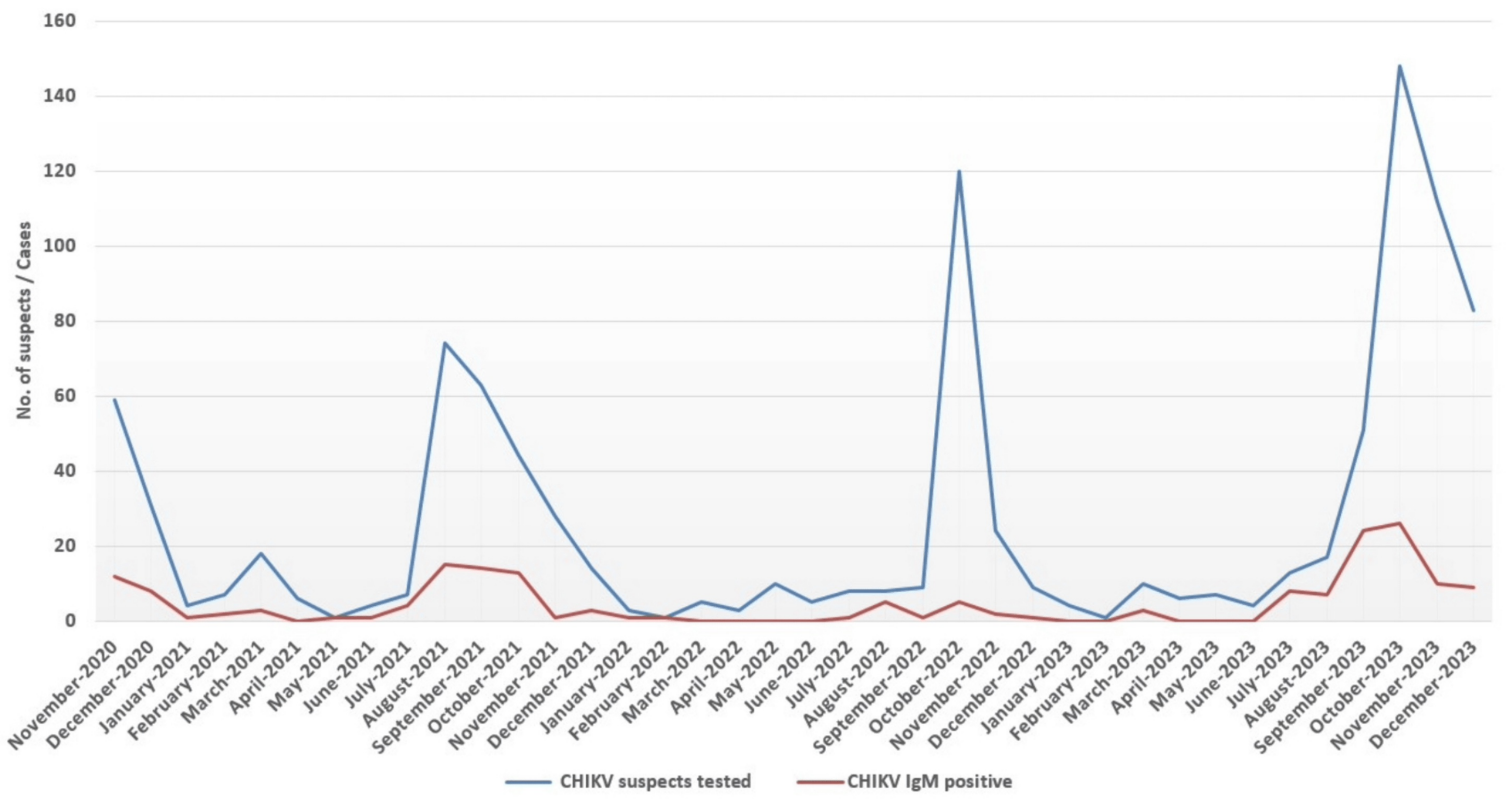
Month-wise distribution of tested CHIKV suspects and CHIKV IgM-positive samples CHIKV: chikungunya virus.

Figure 1shows the month-wise and year-wise distributions of cases, with an increasing trend observed over the years. Among these, 16 samples (9.3%) also tested positive for CHIKV RNA through RT-PCR, confirming an active infection. These RNA-positive samples were subjected to sequencing, yielding an average Q30 of 93.5%, ensuring high-quality data for downstream bioinformatics analysis.

Phylogenetic analysis

**Figure 2:**
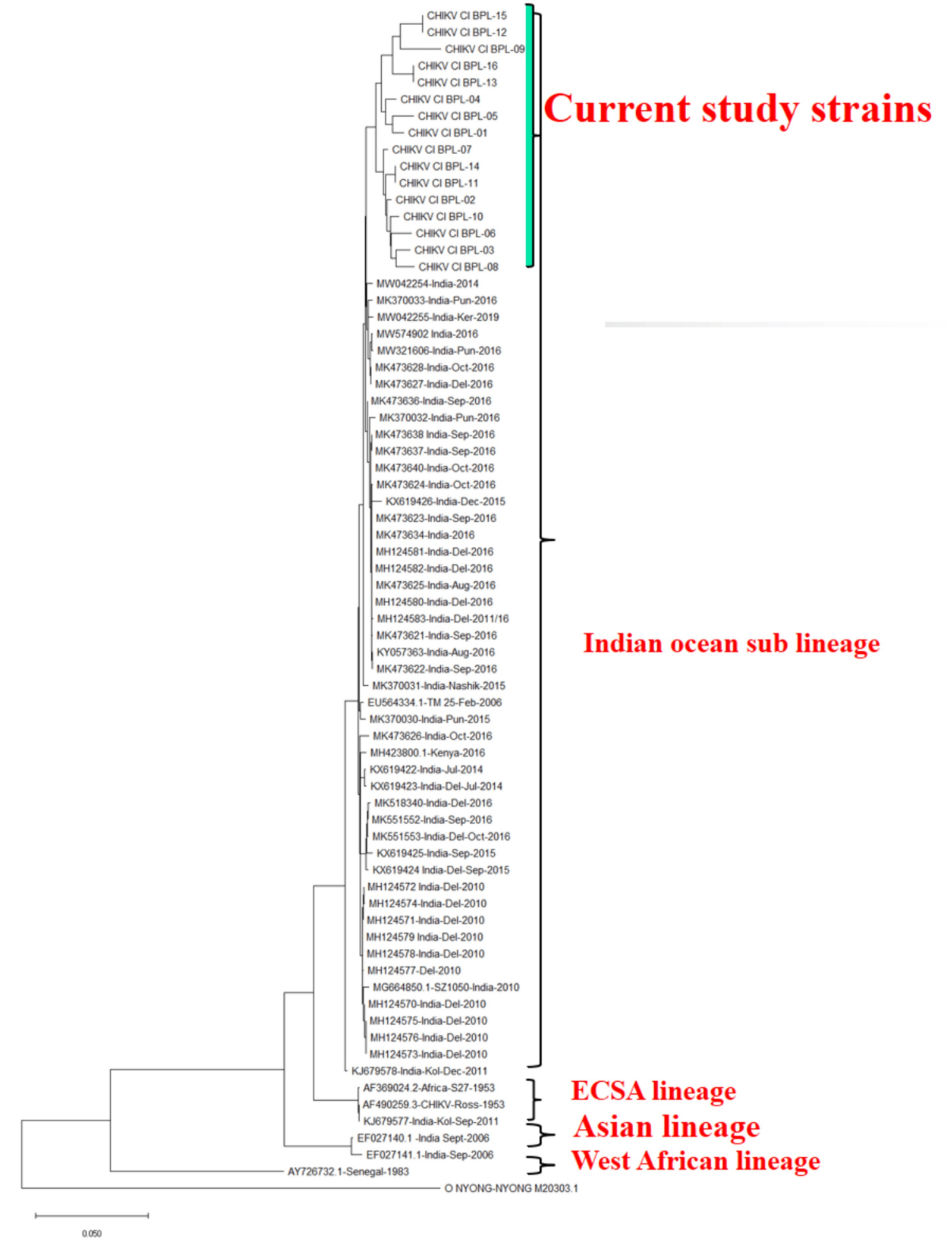
Molecular phylogeny of sequenced CHIKV strains showing their relationship to different lineages and past Indian outbreak strains using the maximum likelihood method by MEGA 11 CHIKV: chikungunya virus. Image Credit: Authors' original creation.

Sequencing of the 16 CHIKV-positive samples revealed that all isolates clustered together and closely resembled the Indian Ocean sublineage within the East-Central-South-African (ECSA) genotype. Phylogenetic analysis showed that the strains in our study were most closely related to CHIKV strains reported from India in 2014, 2016, and 2019. These strains diverged from the ECSA strains of 1953 and later from the 2011 strains of Kolkata, forming a distinct cluster (Figure 2).

Mutational analysis

After obtaining the CHIKV WGS from our region, we aimed to track its molecular evolution by comparing it with previously documented sequences from India.

Notable mutations in the E1 gene included S72N, K211E, M269V, D284E, A315V, and I317V. We also observed some "unstable" mutations, such as M31I, I54V, and S105T, which reverted to previous sequences in the E1 gene. The epidemic-associated mutation A226V, which enhances viral fitness in *Aedes albopictus* mosquitoes, was absent.

Unstable mutations were also observed at two positions in the capsid (C) protein gene, wherein asparagine at positions 11 and 82 was replaced by serine in the 2015 and 2016 strains, but reverted in our strains. Table 2 shows the comparison of such unstable mutations with those reported previously. 

**Table 2:** Unstable mutations in CHIKV observed over the years CHIKV: chikungunya virus.

Gene/protein	Positions	Mutations	Year reported	Year reverted	In the current study
E1	31, 55, 105, 211	M31I, I55V, S105T, K211E	2015, 2016	Reverted 2019 onward	Reverted
E2	57, 72, 79, 135, 143, 181, 195, 284, 287, 299, 312, 344, 375, 386, 390	G57K, N72S, E79G, N135V, S143Y, L181M, Q195R, A284V, N287R, N299S, M312T, A344T, S375T, A386V, M390V	Post-2016	-	Reverted
E3	8, 23, 42	C8S, A23I, I42V	Post-2011	Reverted 2020 onward	Reverted
C	11, 82	N11S, N82S	2015, 2016	Reverted 2019 onward	Reverted
nsP1	184, 288	M184T, V288I	Post-2016	-	Reverted
nsP2	130, 145, 149, 157, 213, 375	H130Y, E145D, G149P, T157I, N213H, E375Q	Post-2016	-	Reverted
nsP3	29, 31, 36, 38, 54, 55, 126, 326, 341, 352, 382, 418, 445, 468, 470	P29A, D31G, A36S, Y38H, T54A, A55T, N126D, S326P, T341M, E352K, T382A, E418K, R445H, F468Y, A470V	Post-2016	-	Reverted
nsP4	55, 85, 424, 555, 604	S55N, K85R, F424L, I555V, I604V	Post-2016	-	Reverted

Some mutations were stable, such as S72N, I317V, nsP2:H130Y, nsP3:Y217H, nsP4:S55N, nsP4:R85G, and nsP4:R245Q. Additionally, mutations such as nsP2-E145D and nsP3-V376T re-emerged in our isolates.

## Discussion

Our study, based on WGS of CHIKV strains, uncovered several noteworthy findings. The demographic and temporal characteristics of CHIKV infections provide valuable insights into the circulating strains and the molecular epidemiology of the disease in our region. Notably, the overall CHIKV IgM antibody positivity rate was 17.4%, with the highest prevalence in the 31-40 age group, which is associated with higher physical activity and stronger immune responses. These individuals may mount a more robust IgM antibody response. However, statistical analysis revealed no significant difference in IgM positivity rates across age groups (p = 0.109), suggesting that any observed variations are likely due to random chance rather than a true age-related effect. Further studies with larger sample sizes are needed to explore this finding in more detail.

An overall increasing trend in suspected and confirmed cases was observed over the years, with month-wise peaks during the monsoon season [21,22]. In contrast, cases consistently declined during the winter and summer months. Year-on-year variations further underscore the unpredictable nature of CHIKV outbreaks, highlighting the challenges in forecasting their magnitude and timing, which are influenced by viral adaptations and evolving epidemiological factors.

Our study uncovered the molecular epidemiology of CHIKV, revealing that all the 16 strains we sequenced clustered in one group alongside the isolates from India in 2014, 2016, and 2019 [23-26]. Among the ECSA genotypes, our strains showed the closest genetic proximity to the outbreaks in New Delhi (2014-2016) and Kerala (2019) [23,27-29]. These findings highlight the persistence of CHIKV clades and their possible local evolution within the Indian Ocean sublineage in Central India.

Our study highlights the dynamic nature of CHIKV mutations, with certain genetic reversions aligning with findings from earlier research. We observed the reversion of mutations such as E1 A226V, M31I, I54V, and S105T, which were widely reported in studies before 2011 [25]. Similar reversions were identified in the C protein, where asparagine at positions 11 and 82, previously replaced by serine in the 2015 and 2016 strains, reverted to their original forms like the 2019 strains [23]. These findings suggest that while some particular mutations may confer a transient survival advantage in certain environmental conditions, there is a possibility that some of these might be unstable and can revert to ancestral forms when evolutionary selective pressures change.

Most importantly, the absence of the A226V mutation, which enhances CHIKV transmission through *Aedes albopictus*, suggests that current strains may be primarily transmitted through *Aedes aegypti*​​*, *consistent with recent findings from Central and North India [24,25,27]. This shift in vector preference underscores the importance of continuous surveillance to monitor potential changes in vector competence and epidemiological trends.

Additionally, our findings reveal the re-emergence of mutations such as nsP2-E145D and nsP3-V376T, which were present in the 2011 isolates but disappeared between 2014 and 2016. Another intriguing observation is that at position 524 of the nsP3 gene, a mutation has replaced the stop codon with the amino acid arginine, which is known to attenuate the clinical severity of arthritis among the affected [25]. It has also re-emerged in our strains and was last reported among the isolates from India in 2011 [30].

Furthermore, the emergence of new mutations such as I317V, which is considered a novel mutation emerging recently, including strains from Central India and New Delhi, suggests a pattern of localized viral evolution [23,24]. This mutation could have functional implications for viral fitness, transmission efficiency, and host pathogenicity, necessitating further investigation.

A key strength of our study is the large-scale screening of over 1,000 samples tested within three years in the post-COVID-19 era. This extensive dataset provides valuable epidemiological insights at a time when the pandemic significantly hampered disease surveillance efforts. Additionally, our findings contribute to the growing body of knowledge on CHIKV’s unpredictable periodicity and diverse clinical manifestations. Our findings could help guide the development of low-cost PCR-based kits targeting specific mutations to differentiate and identify stains circulating locally. However, we acknowledge certain limitations, including the use of only 16 samples for WGS due to the short viremia period and the consequent low RT-PCR positivity rates. Further research is warranted to elucidate the functional significance of these mutations, their underlying mechanisms, and their impact on viral transmission dynamics.

Our findings pave the way forward for studying possible associations between viral genotypes and mutations and clinical characteristics like arthralgia, clinical severity, and duration of illness. Additionally, these insights hold significant value for vaccine candidates in terms of assessing their protective efficacy against locally circulating strains. Ongoing surveillance efforts appear to be of critical importance, particularly in endemic regions, to improve our understanding of this disease, which has unpredictable periodicity and varied clinical manifestations. Similar molecular analysis in mosquito samples can serve as warning signs for impending outbreaks and detect emerging mutations, playing a role in guiding public health interventions and outbreak preparedness strategies.

## Conclusions

This study confirms the circulation of the ECSA genotype of CHIKV in our region and also identifies some key stable and reverting mutations. Additionally, our findings suggest that currently circulating CHIKV strains are primarily transmitted by *Aedes aegypti*, consistent with recent reports from central and northern India. This enhances our understanding of the evolutionary dynamics of local CHIKV clades in Central India. By contributing to the broader picture of CHIKV's genetic diversity, our study underscores the critical need for continuous molecular surveillance of CHIKV's evolution. Such insights are essential for epidemiologic monitoring, guiding effective management and control strategies, and strengthening global public health efforts against chikungunya.

## Appendices

Appendix 1 

**Table 3:** Details of the genome sequences utilized from the NCBI for phylogeny construction NCBI: National Center for Biotechnology Information.

S. no.	Accession	Region	Month and year
1	AF369024.2	Africa-S27	1953
2	AF490259.3	CHIKV-Ross-Africa	1953
3	AY726732.1	Senegal	1983
4	EU564334.1	TM 25	February 2006
5	EF027140.1	India	September 2006
6	EF027141.1	India	September 2006
7	MH124570	Delhi, India	2010
8	MH124571	Delhi, India	2010
9	MH124572	Delhi, India	2010
10	MH124573	Delhi, India	2010
11	MH124574	Delhi, India	2010
12	MH124575	Delhi, India	2010
13	MH124576	Delhi, India	2010
14	MH124577	Delhi	2010
15	MH124578	Delhi, India	2010
16	MH124579	Delhi, India	2010
17	MG664850.1	SZ1050-India	2010
18	KJ679577	Kolkata, India	September 2011
19	KJ679578	Kolkata, India	December 2011
20	MH124583	Delhi, India	2011
21	KX619422	India	July 2014
22	KX619423	Delhi, India	July 2014
23	MW042254	India	2014
24	KX619424	Delhi, India	September 2015
25	KX619425	India	September 2015
26	KX619426	India	December 2015
27	MK370030	Pune, India	2015
28	MK370031	Nashik, India	2015
29	KY057363	India	August 2016
30	MK473625	India	August 2016
31	MK473621	India	September 2016
32	MK473622	India	September 2016
33	MK473623	India	September 2016
34	MK473636	India	September 2016
35	MK473637	India	September 2016
36	MK473638	India	September 2016
37	MK551552	India	September 2016
38	MH124580	Delhi, India	2016
39	MH124581	Delhi, India	2016
40	MH124582	Delhi, India	2016
41	MK473624	India	October 2016
42	MK473626	India	October 2016
43	MK473628	India	October 2016
44	MK473640	India	October 2016
45	MK551553	Delhi, India	October 2016
46	MK518340	Delhi, India	2016
47	MK473627	Delhi, India	2016
48	MK473634	India	2016
49	MW321606	Pune, India	2016
50	MW574902	India	2016
51	MW574902	India	2016
52	MH423800.1	Kenya	2016
53	MK370032	Pune, India	2016
54	MW042255	Kerala, India	2019
55	MW042255	O_nyong-nyong (outlier)	-
